# Cell-free DNA methylomics identify tissue injury patterns in pediatric ARDS

**DOI:** 10.1172/jci.insight.191684

**Published:** 2025-09-02

**Authors:** Nadir Yehya, Jacob E. Till, Nishi Srivastava, Donglan Zhang, Jason D. Christie, Erica L. Carpenter, Nilam S. Mangalmurti, Wanding Zhou

**Affiliations:** 1 Department of Anesthesiology and Critical Care Medicine, Children’s Hospital of Philadelphia and University of Pennsylvania, Philadelphia, Pennsylvania, USA.; 2 Department of Medicine, Perelman School of Medicine, University of Pennsylvania, Philadelphia, Pennsylvania, USA.; 3 Center for Translational Lung Biology, University of Pennsylvania, Philadelphia, Pennsylvania, USA.; 4 Center for Computational and Genomic Medicine, Children’s Hospital of Philadelphia, Philadelphia, Pennsylvania, USA.

**Keywords:** Inflammation, Pulmonology, Biomarkers, Epigenetics

## Abstract

Plasma cell-free DNA can ientify what tissues are damaged in children with severe lung injury, allowing us to identify new avenues to target therapies.

**To the Editor:** Cell-free DNA (cfDNA) is released into plasma from dying cells and is elevated in critical illness syndromes including acute respiratory distress syndrome (ARDS) (1) acting as a damage-associated molecular pattern (DAMP) to propagate inflammation (2). However, the tissue source of circulating cfDNA is unclear. Identifying the cellular origin of cfDNA may provide insight into the mechanisms underlying the inflammatory response. Since cfDNA retains the unique methylation pattern of its tissue of origin (3), we conducted a pilot study leveraging cfDNA methylomics to characterize the spectrum of tissue injury in pediatric ARDS. As a preliminary assessment, we focused on differences between inflammatory subphenotypes.

Additional details are provided in the Supplemental Methods (supplemental material available online with this article; https://doi.org/10.1172/jci.insight.191684DS1). Briefly, this was a secondary analysis of a cohort of children with ARDS (*n* = 333) from the Children’s Hospital of Philadelphia enrolled between 2014 and 2019, with plasma collected within 24 hours of ARDS onset after obtaining prospective caregiver consent.

Since this was the first cfDNA methylomic study in pediatric ARDS, to our knowledge, we purposefully included patients with higher levels of cfDNA to assess feasibility. We randomly selected 24 children with cfDNA levels above the median value (from *n* = 333) and 6 intubated non-ARDS controls. We isolated and quantified plasma cfDNA, and we analyzed the methylome using the Infinium MethylationEPIC (Batch 1, *n* = 8 ARDS) and Epic v2.0 (Batch 2, *n* = 16 ARDS and 6 controls) BeadChips (4). Methylation was deconvoluted by mapping against known tissue methylation patterns to identify cellular origin. Hypo- and hyperinflammatory ARDS were defined using a published algorithm. We compared hypoinflammatory ARDS, hyperinflammatory ARDS, and non-ARDS controls using nonparametric statistics. We also correlated log-transformed cfDNA levels with log-transformed biomarker levels.

Of the 24 children with ARDS (Supplemental Table 1), nonpulmonary sepsis (46%) and pneumonia (38%) were the most common etiologies. Illness severity was high, with 92% of patients requiring vasopressors and 46% being immunocompromised. Of the cohort, 75% were categorized as hyperinflammatory ARDS, and 90-day mortality was 46%.

Hyperinflammatory ARDS had higher cfDNA levels relative to both hypoinflammatory ARDS (rank-sum *P* < 0.05) and non-ARDS controls (*P* < 0.01) (Supplemental Figure 1). t-SNE dimensionality reduction discriminated hypo- from hyperinflammatory ARDS. The dominant signature was leukocytes in all samples (Figure 1). When examining cfDNA levels across cell types, there was generally a stepwise increase from non-ARDS controls (lowest) to hypoinflammatory to hyperinflammatory ARDS (highest) (Supplemental Figures 2–4). Monocytes/macrophages, T cells, endothelial cells, and hepatocytes were significantly different between hypo- and hyperinflammatory ARDS. Hyperinflammatory ARDS had a higher proportion of monocytes (23% hyper versus 15% hypo), B cells (12% versus 9%), and CD8^+^ T cells (19% versus 13%), as well as a lower proportion of granulocytes (19% versus 38%), than hypoinflammatory ARDS (Supplemental Figure 3).

When tested for correlation with biomarkers of inflammation or tissue damage (Supplemental Figure 5), hepatocyte cfDNA was correlated with alanine aminotransferase (ALT), lung epithelial cfDNA correlated with surfactant protein D (SPD), and vascular endothelial cfDNA was correlated with angiopoietin 2 (ANGPT2). There was no correlation between leukocyte or granulocyte cfDNA levels with either total white blood cell (WBC) counts nor with absolute neutrophil counts (ANCs).

Pediatric ARDS, especially hyperinflammatory ARDS, was characterized by elevated plasma cfDNA originating primarily from leukocytes, consistent with leukocyte turnover propagating inflammation by generating additional DAMPs. Plasma cfDNA derived from monocyte/macrophages and CD8^+^ T cells were higher (both in concentration and proportion) in hyperinflammatory ARDS, whereas cfDNA from granulocytes were proportionately lower, suggesting that neutrophils were not the sole driver of hyperinflammation.

Our findings confirm the widespread tissue damage associated with hyperinflammatory ARDS. Interestingly, hyperinflammatory ARDS was also characterized by high levels of hepatocyte and endothelial death. Hepatocyte death may specifically contribute to excessive inflammation by impairing clearance of cfDNAs and other DAMPs (5). The hyperinflammatory subphenotype has greater prevalence and severity of shock, more organ failures, and higher mortality. Our study adds to this by highlighting the significance of endothelial cell death as a potentially targetable organ failure in hyperinflammatory ARDS.

Plasma cfDNA methylomics is a potentially novel approach to characterize tissue injury in critical illness. However, our study has important limitations. First, the sample size is small, and heterogeneity precludes firm conclusions regarding the differences in cellular origins of cfDNA between hypo- and hyperinflammatory ARDS. Second, we used arrays with limited coverage. Whole genome alternatives, such as deep bisulfite sequencing, are more precise (3). Third, the original EPIC array was discontinued during the course of this study, necessitating use of EPIC v2.0, with risk of batch effect. However, our benchmarking (4) and correlation with biomarkers reassure us that the differences are modest and unlikely to confound the main biological signals in our analysis. Additionally, our reported percentages of leukocyte subtypes approximate what was reported for adults with COVID-19 (6). Finally, as this is a rapidly evolving field, tissue assignments are subject to revision (7). We anticipate that future studies using cfDNA methylomics will employ newer techniques and updated libraries to more precisely quantify cell damage, thereby validating and building upon these initial findings.

## Supplementary Material

Supplemental data

Supporting data values

## Figures and Tables

**Figure 1:**
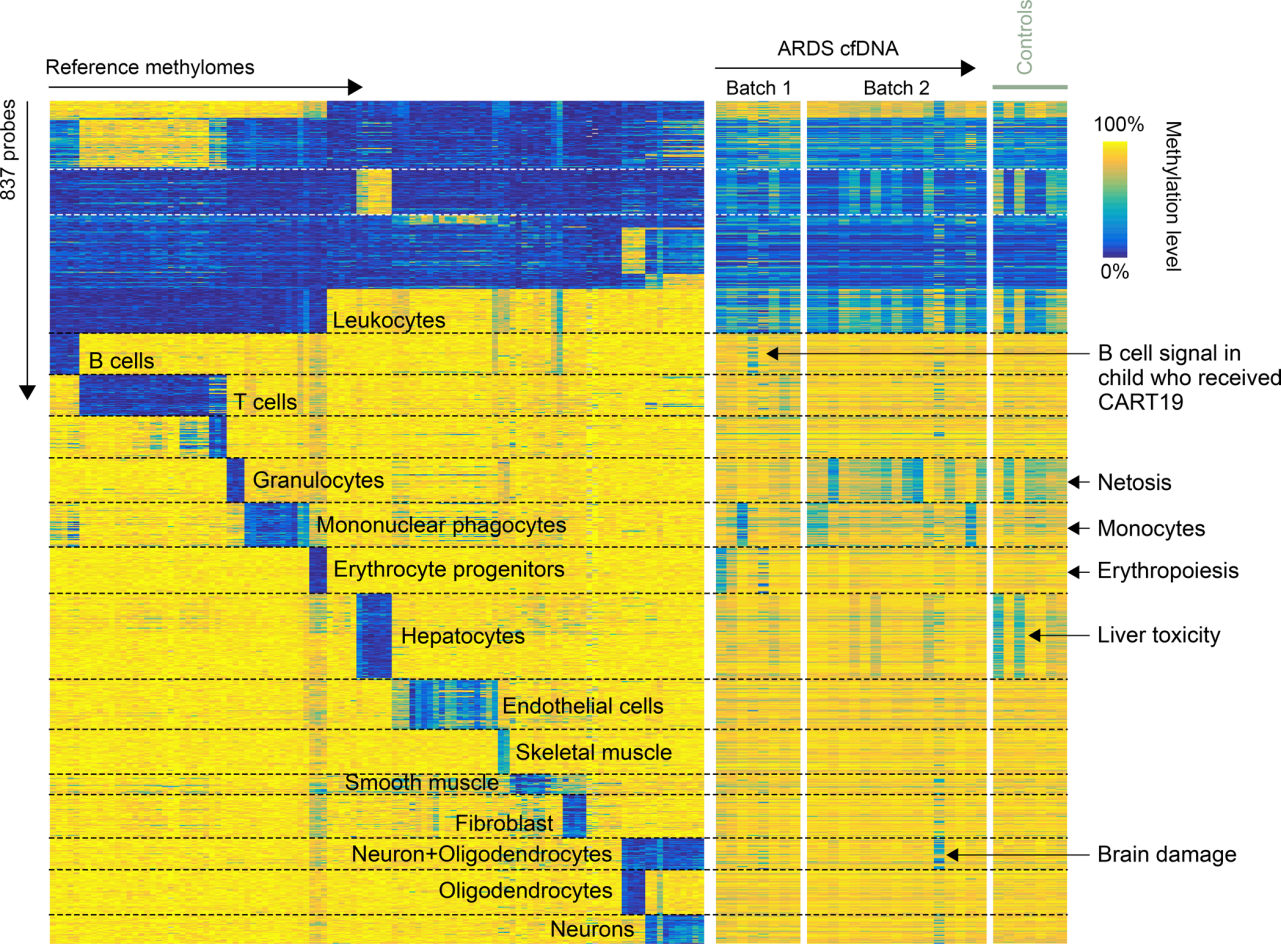
Comparing methylation (837 CpG probes) of isolated reference cells, ARDS, and controls. Myeloid cells dominate most profiles, with 2 distinct subgroups: a larger group with mainly granulocytes (potentially NETosis) and a smaller group enriched for monocyte. In 1 CART19-treated patient, a B cell cfDNA signal was observed, consistent with CART-19 targeting B cells. Several patients with ARDS and controls displayed signal from liver cells, suggesting liver damage. A neuron/glial cell signal was detected in 1 sample, potentially reflecting brain injury, and 1 sample exhibited a strong signal from erythrocyte progenitors.

## Version 1 09/02/2025

In-Press Preview

## Version 2 10/22/2025

Electronic publication
