# Supplementary material for: Cell-free DNA methylomics identify tissue injury patterns in pediatric ARDS
Source: JCI Insight. 2025 Sep 2;10(20):e191684. doi: 10.1172/jci.insight.191684 (PMC12581656; doi:10.1172/jci.insight.191684)
Supplement: Supplemental data [file jciinsight-10-191684-s061.pdf]

Cell-free DNA Methylomics Identify Tissue Injury Patterns in Pediatric ARDS

DATA SUPPLEMENT

Supplementary Methods	2
Supplementary Table 1: Demographics	8
Supplementary Figure 1: cfDNA levels between ARDS subphenotypes	9
Supplementary Figure 2: cfDNA levels of blood cells	10
Supplementary Figure 3: Proportion of leukocytes between subphenotypes	11
Supplementary Figure 4: cfDNA levels of other cell types	12
Supplementary Figure 5: Correlation between cfDNA and tissue markers	13
References	14

SUPPLEMENTARY METHODS

Overview of the Larger Cohort

This was a secondary analysis of a subset of 333 patients from a prospective cohort study of children with Berlin-defined (1) acute respiratory distress syndrome (ARDS) enrolled at the Children's Hospital of Philadelphia (CHOP) between July 2014 and December 2019. The overall aim of this larger cohort study was to associate select biomarkers with clinical outcomes, with a pilot phase of sample collection only on day 0 (≤ 24 hours of ARDS onset)(2, 3) and a subsequent longitudinal phase with sample collection on days 0, 3, and 7. The cfDNA methylomic sub-analysis has not been previously reported.

PICU patients were screened daily. Inclusion criteria were 1) acute respiratory failure requiring invasive ventilation, 2) arterial access, 3) age > 1 month and < 18 years, 4) two consecutive $PaO_2/FIO_2 \leq 300 \geq 1$ hour apart on positive end-expiratory pressure (PEEP) ≥ 5 cmH_2O, and 5) bilateral infiltrates separately adjudicated by a radiologist and intensivist. Exclusion criteria were 1) respiratory failure primarily from cardiac failure, 2) chronic respiratory disease, 3) ventilator dependence, 4) cyanotic heart disease, 5) ventilation for > 7 days before $PaO_2/FIO_2 \leq 300$, and 6) ARDS established outside of the CHOP PICU.

For comparison, we also enrolled 25 intubated children with ARDS risk factors screened for the ARDS cohort, but not meeting oxygenation ($PaO_2/FIO_2 > 300$) or radiographic criteria, into the larger parent study as non-ARDS controls. From this cohort, we selected the 6 subjects with highest levels of cfDNA (3 with viral lower respiratory tract infections and 3 meeting Sepsis 3 criteria)(4). Controls were not matched on age, diagnosis, or illness severity in this pilot study.

Definitions

ARDS was characterized as direct (primarily pulmonary) and indirect (primarily non-pulmonary). Infectious pneumonia, aspiration, drowning, pulmonary contusion, and smoke inhalation were considered direct ARDS; non-pulmonary sepsis, non-thoracic trauma, non-cardiogenic shock, transfusion-related acute lung injury, and pancreatitis were indirect. Etiology was determined primarily by chart abstraction by trained study personnel in discussion with the attending physician on the likely etiology. Uncertain cases were adjudicated by a three-person team of PICU physicians, with discussion until unanimous consensus. Assignment of subjects to hypo- and hyperinflammatory ARDS subphenotypes was performed using parsimonious algorithms described for this cohort (5).

Metrics of oxygenation utilized were PaO_2/FIO_2 and oxygenation index (mean airway pressure x FIO_2 x 100)/PaO_2). Shock severity was quantified with the vasopressor score (6, 7). The designation "immunocompromised" required presence of an immunocompromising diagnosis (oncologic, immunologic, rheumatologic, transplant) and active immunosuppressive therapy, or presence of a congenital immunodeficiency (8, 9).

Sample Processing

Blood was collected in citrated tubes within 24 hours of ARDS onset (time of meeting all Berlin criteria). Samples were centrifuged (2000 g, 20 minutes, 20C) within 30 minutes of sample collection, aliquoted to prevent freeze/thaw cycles, and stored at -80C until analysis. Circulating cfDNA was extracted from a 250 µL plasma aliquot using the DNeasy Blood and Tissue kit (Qiagen). DNA levels were quantified in triplicate using a LightCycler Fast Start DNA

Master SYBR green I (Roche) and QuantStudio 7 (Applied Biosystems) qPCR for a 115 bp amplicon of the human ALU repeat (10). As part of a separate analysis (5), angiopoietin-2 (ANGPT2), the soluble receptor for advanced glycation end-products (sRAGE), and surfactant protein D (SPD) were measured by ELISA in duplicate from the same plasma samples as cfDNA.

Methylomic Measurements and Analyses

We isolated and quantified cfDNA from plasma, and performed methylation analysis using the Infinium MethylationEPIC and MethylationEPIC v2.0 BeadChip (11). We used 10 ng input DNA for a pre-amplification step, as we have reported previously (12), and loaded 500 ng input onto the array. Processing IDAT files generated from Infinium BeadChip-based data was performed using the *openSesame* workflow within the *SeSAMe* package (13). This workflow effectively addresses critical processing challenges, including signal background correction, dye bias adjustment, inference of color channel swaps, masking of suboptimal signals, and precise extraction of DNA modification levels (beta values). To ensure data quality and integrity, tissue type, ethnicity, sex, and copy number were verified using specialized functions within *SeSAMe*, allowing for the identification and resolution of potential sample mix-ups (14). The processed data were visualized using t-SNE projections to assess overall sample clustering and quality. For subsequent analyses, the data were structured as a cytosine methylation matrix, with rows representing CpG sites and columns corresponding to individual samples or cell types.

Methylation patterns were deconvoluted by mapping against known cell methylation patterns to identify tissues of origin. The algorithm employs a stochastic simulated annealing approach for cell-type deconvolution using DNA methylation data. The algorithm and reference

data are implemented in the CytoMethIC package (model ID: TissueComp_EPIC_20240717 and TissueComp_EPICv2_20240717), with the reference panel based on previously published cell-type-resolved whole-genome methylation profiles (15). The feature selection begins by selecting CpGs with the largest differences in methylation (delta beta) between target and non-target cell types, focusing on hypomethylation. This constructs a reference methylation profile for each cell type using the top-ranked features. The deconvolution process estimates cell-type proportions by minimizing the error between the observed methylation levels and a weighted combination of these reference profiles. Optimization occurs in two rounds: an initial coarse-grained step involving larger adjustments, followed by a fine-tuned step with smaller refinements to improve accuracy. Random sampling iteratively adjusts cell type proportions by exploring candidate solutions, while probabilistic acceptance of suboptimal solutions prevents the algorithm from becoming trapped in local minima. This approach balances exploration and exploitation to provide robust and accurate estimates of cell-type fractions in complex samples.

Methods Figure 1: Histogram of methylation detection rates, stratified by EPIC version 1 and 2. All samples achieved methylation rates > 80% (> 94% for EPIC v2). Differences in performance between EPIC and EPIC v2 are due to improved probe design and hybridization chemistry (11).

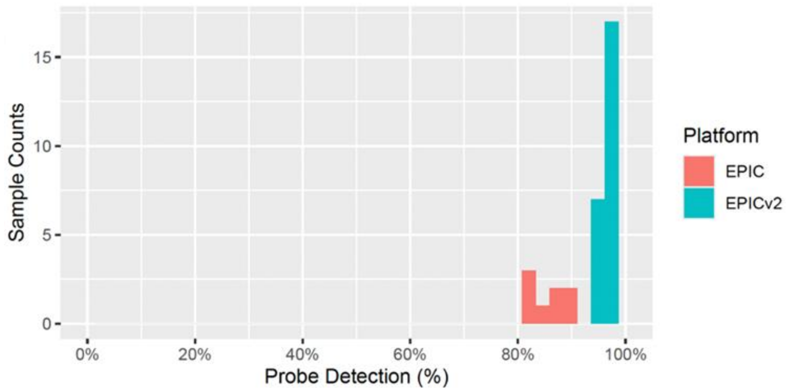

Methods Figure 2: Comparison of EPIC and EPIC v2 samples, with suggestion of batch effect.

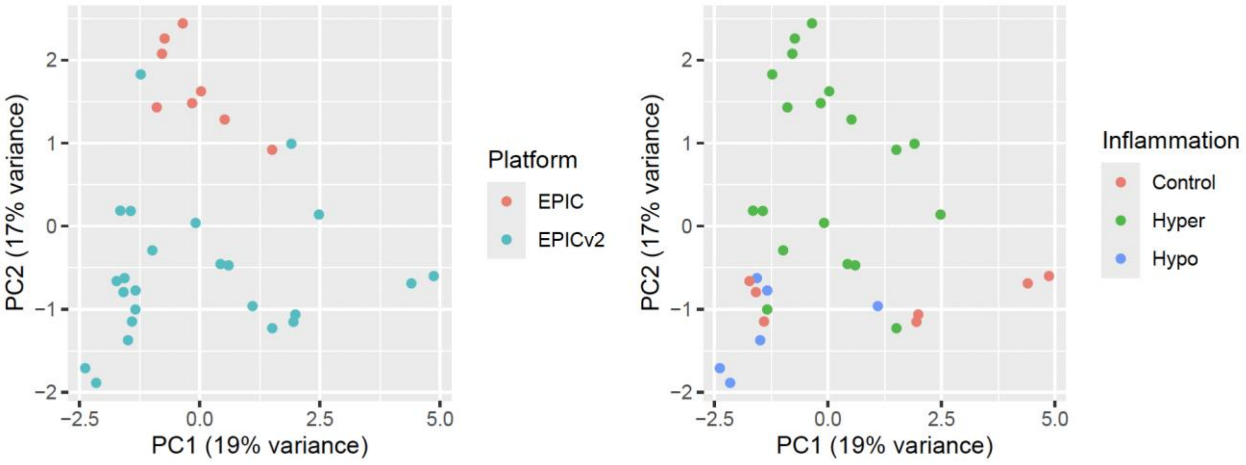

Methods Figure 3: cfDNA beta-values, per sample, demonstrating bimodal distribution.

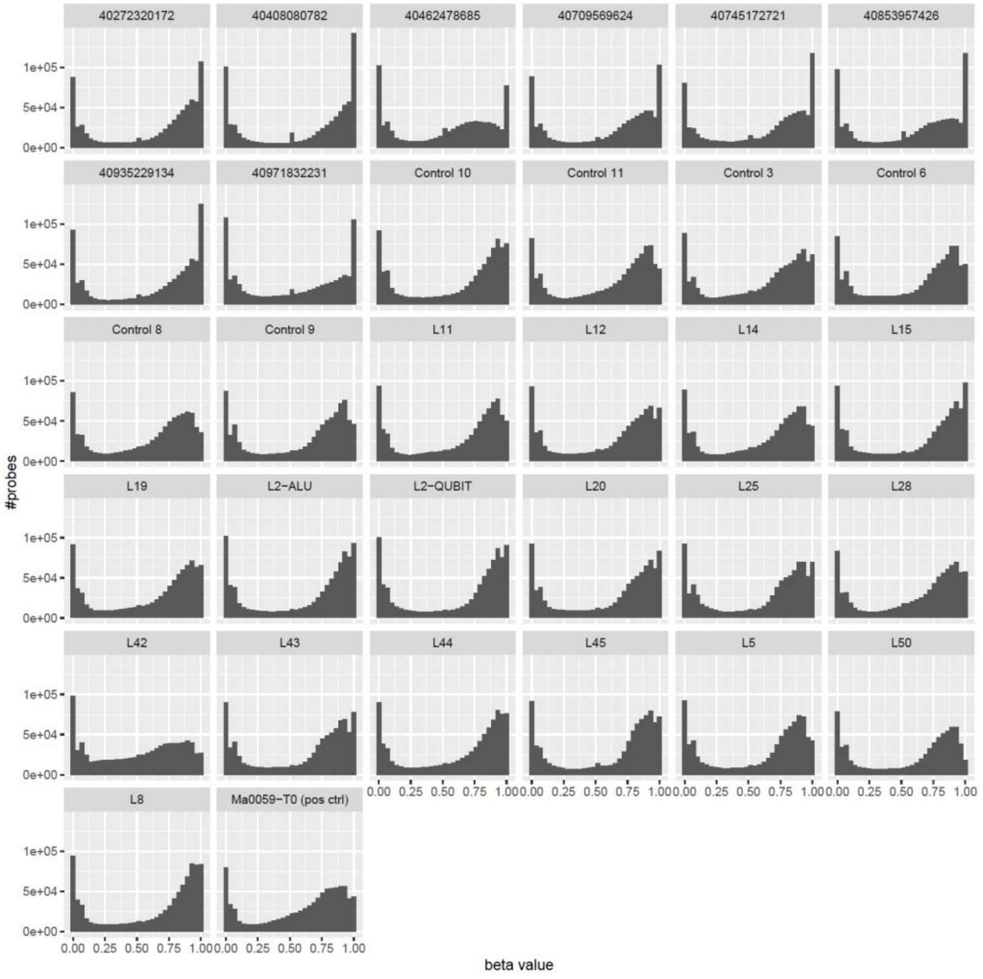

Sex as a Biological Variable

Male and female subjects were included in this study, and 50% of the cohort used in this study was female. In the parent cohort, 44% of subjects were female.

Study Approval

The study was approved by the CHOP Institutional Review Board (IRB 13-010578), and informed consent was obtained from caregivers prior to any study procedures.

Data Availability

Deidentified patient data is available from *JCI Insight* as part of this manuscript submission, and can also be obtained upon request from the corresponding author (Dr. Yehya; yehyan@chop.edu), pursuant to regional legal and regulatory constraints. This is most commonly done with the execution of a data use agreement (DUA) between CHOP and the requesting institution for deidentified (no identifiable health information) data. Deidentified data have been uploaded to the Gene Expression Omnibus (GSE287137). Statistical code is similarly available upon request.

SUPPLEMENTARY TABLE 1: Demographics of the ARDS cohort (n = 24)

**Variables**	**Median [IQR] or N (%)**
Demographics	
Age (years)	8.7 [2.9, 11.7]
Sex (% female)	12 (50)
Severity of illness	
Vasopressor (%)	22 (92)
≥ 2 concurrent vasopressors	11 (46)
Vasopressor score	12 [5, 36]
Co-morbidities	
Immunocompromised (%)	11 (46)
Stem cell transplant (%)	6 (25)
Etiology (%)	
Pneumonia	9 (38)
Non-pulmonary sepsis	11 (46)
Other[a]	4 (17)
Severity of ARDS	
PaO_2/FIO_2	175 [96, 230]
Oxygenation index	12.4 [7.5, 21.1]
ΔP (PIP minus PEEP)[b]	23 [19, 28]
Subphenotype (%)	
Hypoinflammatory	18 (75)
Hyperinflammatory	6 (25)
90-day mortality (%)	11 (46)

[a] One each of aspiration, CART-19, pancreatitis, and drowning.
[b] PIP = peak inflating pressure; PEEP = positive end-expiratory pressure

SUPPLEMENTARY FIGURE 1: (Left) Plasma cfDNA levels between controls, hypo-, and hyperinflammatory ARDS. Overall Kruskal-Wallis p-value is shown, and pairwise comparisons reflect results of Wilcoxon rank-sum test (*: p < 0.05; **: p < 0.01; ***: p < 0.001). (Right) Dimensionality reduction using tSNE showing separation of hypo- and hyperinflammatory ARDS samples.

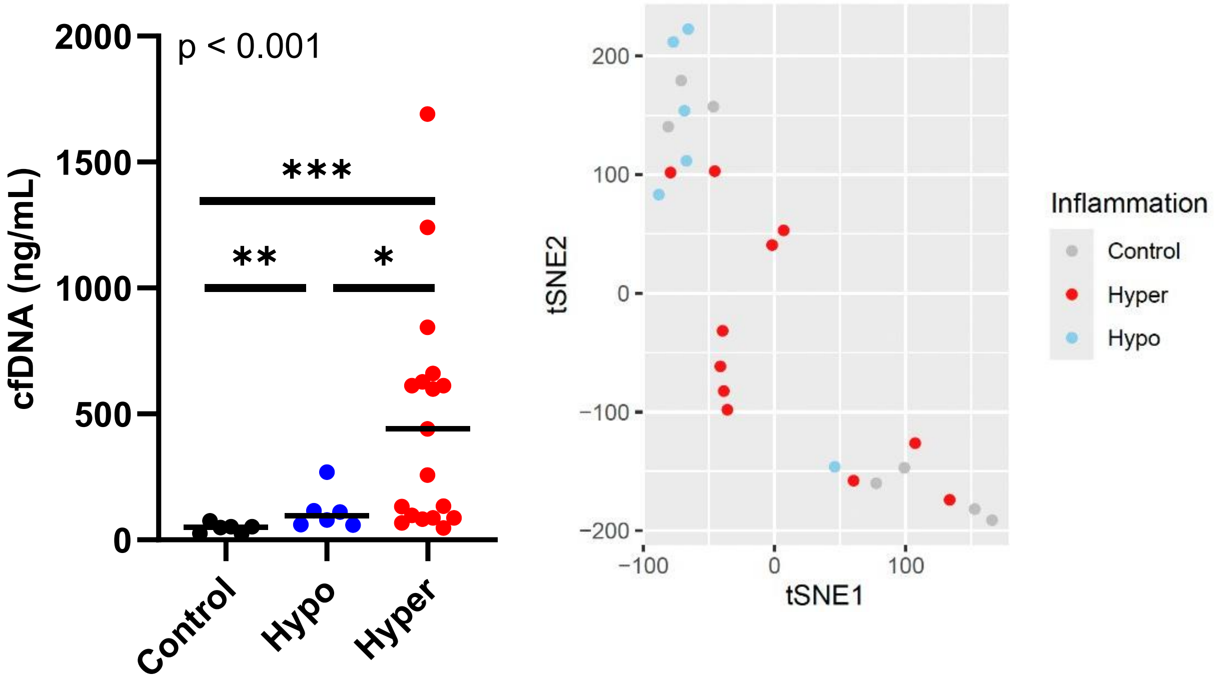

SUPPLEMENTARY FIGURE 2: Plasma cfDNA levels derived from hematopoietic cell lineages between controls, hypo-, and hyperinflammatory ARDS. Overall Kruskal-Wallis p-value is shown, and pairwise comparisons reflect results of Wilcoxon rank-sum test (*: p < 0.05; **: p < 0.01; ***: p < 0.001).

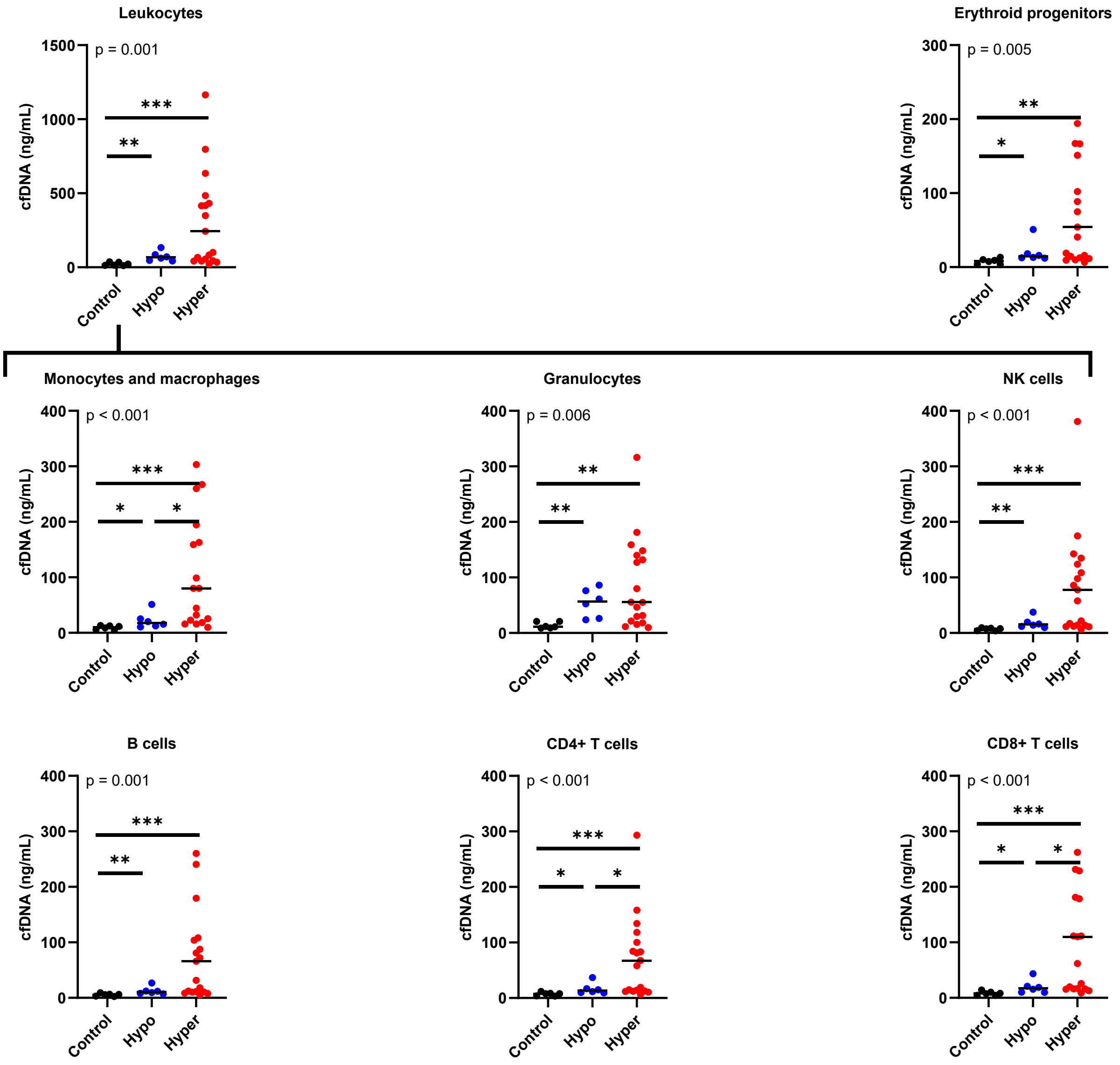

SUPPLEMENTARY FIGURE 3: (Top) Proportions of leukocytes mapped by cfDNA methylomics. Hyperinflammatory ARDS has a higher proportion of monocytes, B cells, and CD8+ T cells, and a lower proportion of granulocytes, than hypoinflammatory ARDS. (Bottom) Proportion of leukocyte cfDNA between controls, hypo-, and hyperinflammatory ARDS. Overall Kruskal-Wallis p-value is shown, and pairwise comparisons reflect results of Wilcoxon rank-sum test.

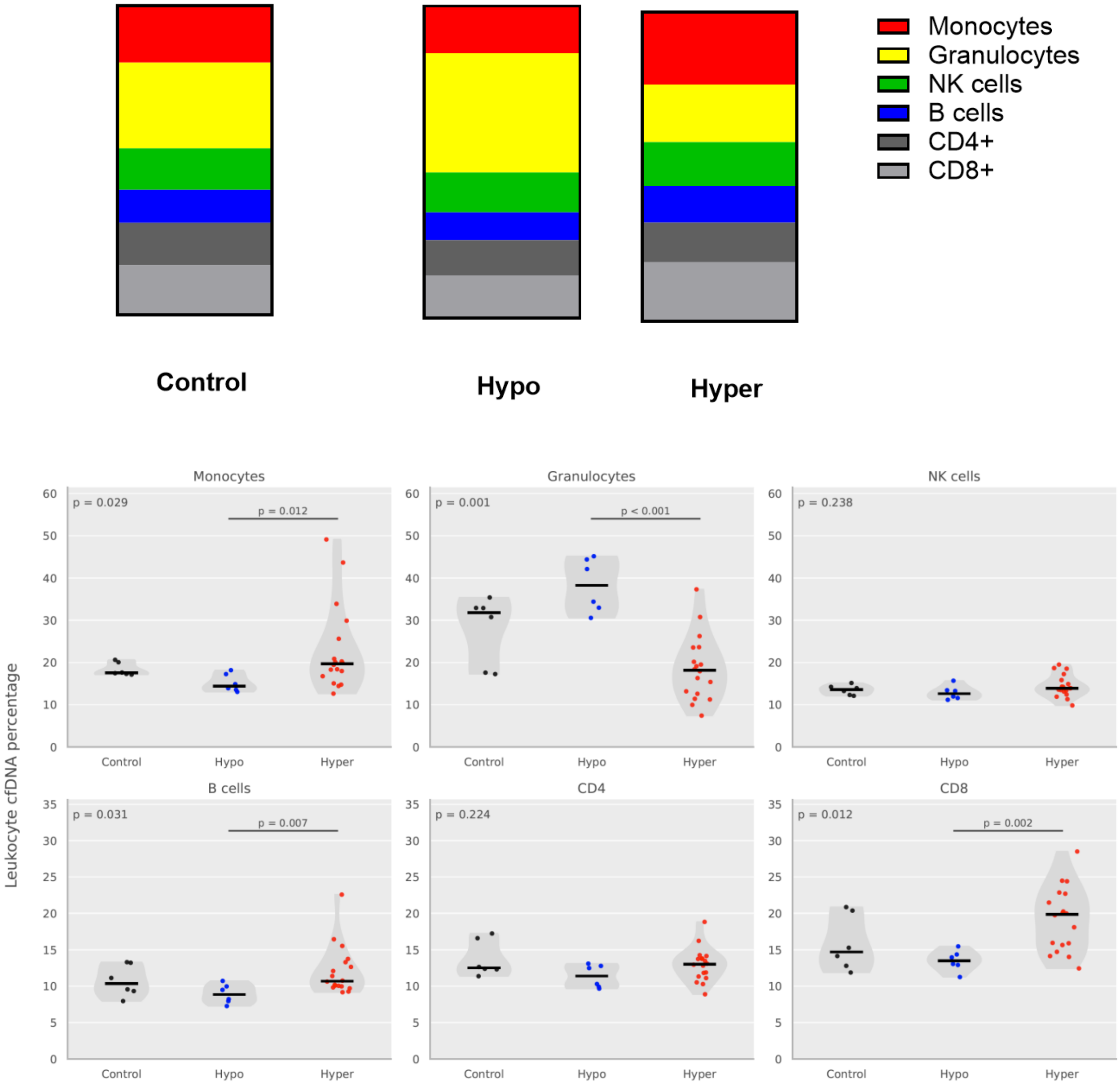

SUPPLEMENTARY FIGURE 4: Plasma cfDNA levels derived from various tissue types between controls, hypo-, and hyperinflammatory ARDS. Overall Kruskal-Wallis p-value is shown, and pairwise comparisons reflect results of Wilcoxon rank-sum test (*: $p < 0.05$; **: $p < 0.01$; ***: $p < 0.001$).

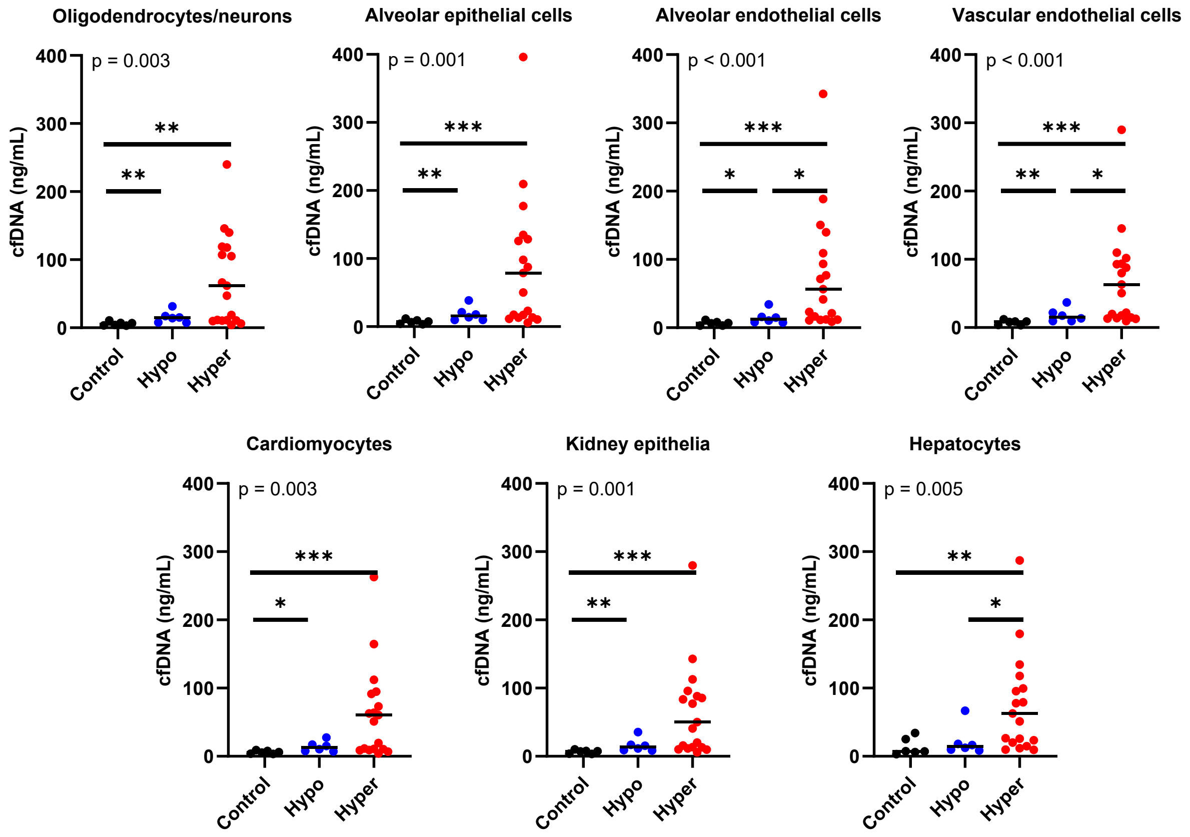

SUPPLEMENTARY FIGURE 5: Correlation plots of plasma cfDNA levels from various tissues with other markers of inflammation or organ damage. Log-transformed cfDNA levels were plotted against log-transformed biomarker levels (cfDNA levels are all on the x-axis). Pearson r and p values are provided.

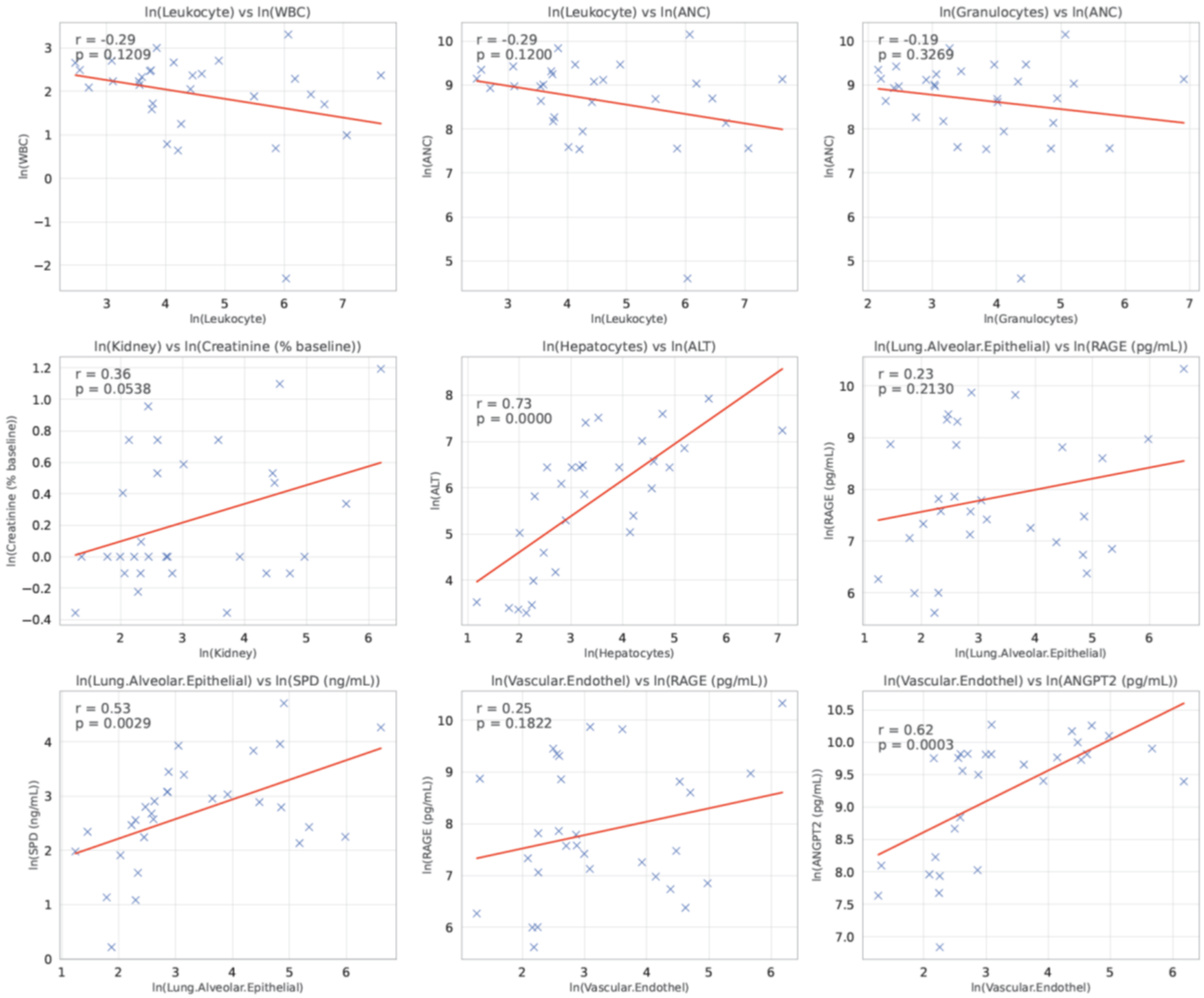

REFERENCES

1. Force ADT, et al. Acute respiratory distress syndrome: the Berlin Definition. *JAMA.* 2012;307(23):2526-33.
2. Yehya N, et al. Circulating nucleosomes are associated with mortality in pediatric acute respiratory distress syndrome. *American journal of physiology Lung cellular and molecular physiology.* 2016;310(11):L1177-84.
3. Yehya N, et al. Circulating markers of endothelial and alveolar epithelial dysfunction are associated with mortality in pediatric acute respiratory distress syndrome. *Intensive Care Med.* 2016;42(7):1137-45.
4. Singer M, et al. The Third International Consensus Definitions for Sepsis and Septic Shock (Sepsis-3). *JAMA.* 2016;315(8):801-10.
5. Yehya N, et al. Identification of molecular subphenotypes in two cohorts of paediatric ARDS. *Thorax.* 2024;79(2):128-34.
6. Wernovsky G, et al. Postoperative course and hemodynamic profile after the arterial switch operation in neonates and infants. A comparison of low-flow cardiopulmonary bypass and circulatory arrest. *Circulation.* 1995;92(8):2226-35.
7. Gaies MG, et al. Vasoactive-inotropic score as a predictor of morbidity and mortality in infants after cardiopulmonary bypass. *Pediatr Crit Care Med.* 2010;11(2):234-8.
8. Yehya N, et al. Improved oxygenation 24 hours after transition to airway pressure release ventilation or high-frequency oscillatory ventilation accurately discriminates survival in immunocompromised pediatric patients with acute respiratory distress syndrome*. *Pediatr Crit Care Med.* 2014;15(4):e147-56.
9. Yehya N, et al. Characterizing degree of lung injury in pediatric acute respiratory distress syndrome. *Crit Care Med.* 2015;43(5):937-46.
10. Fawzy A, et al. Quantitative analysis of plasma cell-free DNA and its DNA integrity in patients with metastatic prostate cancer using ALU sequence. *J Egypt Natl Canc Inst.* 2016;28(4):235-42.
11. Kaur D, et al. Comprehensive Evaluation of The Infinium Human MethylationEPIC v2 BeadChip. *Epigenetics Commun.* 2023;3(1).
12. Lee SM, et al. Low-input and single-cell methods for Infinium DNA methylation BeadChips. *Nucleic Acids Res.* 2024;52(7):e38.
13. Zhou W, et al. SeSAMe: reducing artifactual detection of DNA methylation by Infinium BeadChips in genomic deletions. *Nucleic Acids Res.* 2018;46(20):e123.
14. Zhou W, et al. Comprehensive characterization, annotation and innovative use of Infinium DNA methylation BeadChip probes. *Nucleic Acids Res.* 2017;45(4):e22.
15. Loyfer N, et al. A DNA methylation atlas of normal human cell types. *Nature.* 2023;613(7943):355-64.
